# Inflammation-Driven Reprogramming of CD4^+^Foxp3^+^ Regulatory T Cells into Pathogenic Th1/Th17 T Effectors Is Abrogated by mTOR Inhibition *in vivo*

**DOI:** 10.1371/journal.pone.0035572

**Published:** 2012-04-24

**Authors:** Ekaterina Yurchenko, Marina T. Shio, Tony C. Huang, Maria Da Silva Martins, Moshe Szyf, Megan K. Levings, Martin Olivier, Ciriaco A. Piccirillo

**Affiliations:** 1 Departments of Microbiology and Immunology and Medicine, McGill University, Montreal, Quebec, Canada; 2 Department of Pharmacology, McGill University, Montreal, Quebec, Canada; 3 Child and Family Research Institute, Department of Surgery, University of British Columbia, Vancouver, British Colombia, Canada; 4 FOCIS Center of Excellence, Research Institute of the McGill University Health Center, Montreal, Quebec, Canada; New York University, United States of America

## Abstract

While natural CD4^+^Foxp3^+^ regulatory T (nT_REG_) cells have long been viewed as a stable and distinct lineage that is committed to suppressive functions *in vivo*, recent evidence supporting this notion remains highly controversial. We sought to determine whether Foxp3 expression and the nT_REG_ cell phenotype are stable *in vivo* and modulated by the inflammatory microenvironment. Here, we show that Foxp3^+^ nT_REG_ cells from thymic or peripheral lymphoid organs reveal extensive functional plasticity *in vivo*. We show that nT_REG_ cells readily lose Foxp3 expression, destabilizing their phenotype, in turn, enabling them to reprogram into Th1 and Th17 effector cells. nT_REG_ cell reprogramming is a characteristic of the entire Foxp3^+^ nT_REG_ population and the stable Foxp3^NEG^ T_REG_ cell phenotype is associated with a methylated *foxp3* promoter. The extent of nT_REG_ cell reprogramming is modulated by the presence of effector T cell-mediated signals, and occurs independently of variation in IL-2 production *in vivo*. Moreover, the gut microenvironment or parasitic infection favours the reprogramming of Foxp3^+^ T_REG_ cells into effector T cells and promotes host immunity. IL-17 is predominantly produced by reprogrammed Foxp3^+^ nT_REG_ cells, and precedes Foxp3 down-regulation, a process accentuated in mesenteric sites. Lastly, mTOR inhibition with the immunosuppressive drug, rapamycin, stabilizes Foxp3 expression in T_REG_ cells and strongly inhibits IL-17 but not RORγt expression in reprogrammed Foxp3^−^ T_REG_ cells. Overall, inflammatory signals modulate mTOR signalling and influence the stability of the Foxp3^+^ nT_REG_ cell phenotype.

## Introduction

Naturally-occurring regulatory T (nT_REG_) cells are a central component of peripheral tolerance as they maintain normal immune homeostasis [1], [2], [3]. Originally defined by high expression of CD25 (α subunit of the IL-2 receptor) [4], nT_REG_ cells are now primarily characterized by expression of the transcription factor Foxp3 [5], [6], [7], [8]. Stable expression of Foxp3 is essential for the development, homeostasis and suppressive function of nT_REG_ cells [8], [9]. Functional abrogation of Foxp3 in mice (scurfy mice) and humans (IPEX syndrome) leads to the development of lethal multi-organ autoimmune and inflammatory diseases [10], [11], [12]. On the other hand, forced expression of Foxp3 in conventional CD4^+^CD25^−^ T cells is sufficient for the acquisition of suppressive activity *in vitro* and *in vivo* [5], [6], [7], [13]. Furthermore, the analysis of GFP knock-in, Foxp3 deficient mice revealed that the absence of Foxp3 protein abrogates nT_REG_ suppressive activity, but is dispensable for the maintenance of nT_REG_ cell anergic phenotype [14].

The notion that Foxp3^+^ T_REG_ cells represent a stable, terminally-differentiated lineage has been recently addressed. The transient nature of Foxp3 expression in induced T_REG_ (iT_REG_) cells *in vitro* [15] combined with the potential of Foxp3^+^ nT_REG_ cells to differentiate into Th17 cells in the presence of IL-6 and TGF-β [16], [17], [18] suggested that Foxp3-expressing T_REG_ cell subsets manifest functional adaptation under certain conditions at least *in vitro*. Recent studies reported the down-regulation of Foxp3 expression in nT_REG_ cells *in vivo* under lymphopenic conditions [19], [20], [21] or in organ-specific autoimmunity [22]. Although these reports highlight the functional plasticity of the Foxp3^+^ nT_REG_ cell lineage, more recent studies argue against this phenomenon and propose that Foxp3^+^ T_REG_ cells are refractory to this functional reprogramming [23], [24]. As T_REG_ cell-based therapy is proposed as a treatment in autoimmune and transplantation settings, it is critical to assess the stability of Foxp3^+^ T_REG_ cells and dynamics of there function or reprogramming under inflammatory conditions.

Recent evidence clearly identifies the mammalian target of rapamycin (mTOR), a conserved serine-threonine protein kinase inhibited by the immunosuppressive drug rapamycin, as a master metabolic regulator that integrates environmental cues from nutrients, growth factors and stress conditions to drive cell growth, proliferation and T cell differentiation. More specifically, inhibition of mTOR signalling, either through gene deficiency or rapamycin treatment, promotes T_REG_ differentiation [25], [26], [27] while blunting Th17 differentiation and function *in vitro* and *in vivo* [28]. Currently, the role of mTOR signalling in the process of nT_REG_ cell reprogramming is not well understood.

In this study, we show that the stability of Foxp3^+^ nT_REG_ cell phenotype and function is a dynamic process modulated by inflammatory signals. Thymic or peripheral Foxp3^+^ nT_REG_ cells manifest prominent functional plasticity and readily reprogram into Th1 and Th17 effector cells, particularly in the gut microenvironment or sites of parasitic infection. nT_REG_ cell reprogramming is a characteristic of the entire Foxp3^+^ nT_REG_ population and the stable, reprogrammed T_REG_ cell phenotype is associated with a methylated *foxp3* promoter. The extent of nT_REG_ cell reprogramming is modulated by effector T (T_EFF_) cell-mediated signals but occurs independently of IL-2 dose variations *in vivo*. Reprogrammed Foxp3^+^ T_REG_ cells predominantly produce IL-17, the expression of which precedes Foxp3 down-regulation, a process promoted in the intestinal microenviroment. Lastly, we report a mechanism whereby mTOR inhibition by rapamycin, stabilizes Foxp3 expression and prevents nT_REG_ cell reprogramming into Th1/Th17 effectors *in vivo*. Thus, inflammatory signals may modulate mTOR function and impact the stability of the Foxp3^+^ T_REG_ cell phenotype.

## Materials and Methods

### Mice

WT, TCRβ^−/−^ and Ly5.1 congenic C57BL/6 mice were obtained from Taconic Laboratories, GFP transgenic C57BL/6 (GFPtg) mice were provided by C. Schaefer [29], Foxp3^GFPki^ mice were obtained from A. Rudensky (Memorial Sloan-Kettering Cancer Center) and Ly5.1 Foxp3^GFPki^ congenic line were generated in our laboratory. All mice were bred and maintained in a specific pathogen-free animal facility at McGill University.

### T cell subsets purification and adoptive transfer

Various CD4^+^ T cell subsets were separated from AutoMACS-enriched CD4^+^ T cells using a FACSAria™ Cell Sorter (BD) (purity >98%) or the autoMACS Cell Sorter (Miltenyi Biotec) (purity ranging 85–95%), as described previously [30]. For adoptive transfer studies, T cell subsets were transferred i.v. into TCRβ^−/−^ or RAG2^−/−^ recipient mice. For adoptive transfer studies, T cell subsets were transferred *i.v.* into TCRβ^−/−^ or RAG2^−/−^ recipient mice.

### Lymphocyte isolation from lamina propria

To isolate lymphocytes from lamina propria (LP), a protocol from Weigmann *et al.* was used [31].

### Antibodies and flow cytometry

For surface phenotyping the following mAbs were used: anti-CD4 (RM4–5), anti-CD25 (PC61), anti-CD3 (145-2C11) and anti-Ly5.1 (A120) (eBioscience or BD Bioscience). The expression of Foxp3 (PJK-16s) (e-Bioscience) and Ki-67 (B56) (BD Bioscience) was determined by intracellular staining performed according to the manufactures protocol (e-Bioscience). To determine the cytokine production, lymphocytes were re-stimulated for 4 hrs at 37°C with PMA (20 ng/ml), ionomycin (1 nM) and BD GolgyStop™ (1∶1000 dilution) and then stained intra-cellular with anti-IFN-γ (XMG1.2), anti-IL-17 (TC11-18H10.1 or eBio17B7), anti-IL-10 (JES5-16E3), anti-IL-2 (JES6-5H4) (purchased from eBioscience or BD Bioscience) as described above. Data was acquired on FACSCanto (Becton Dickinson, Mountain View, CA) and analysed using FlowJo software (Tree Star).

### In vivo therapy

*In vivo* IL-2 treatment was performed by *i.p.* injection of 5, 10 or 50 ng of recombinant human IL-2 (rhIL-2) (a kind gift from the Surgery Branch, NCI). *In vivo* rapamycin treatment (0.8 mg/kg) (Sigma) was performed by every other day *i.p.* injection starting one day post T cell-adoptive transfer.

### In vitro T cell functional assay

For *in vitro* proliferation, 5×10^4^ FACS-sorted T cells were plated with 2×10^5^ irradiated total splenocytes and activated with soluble anti-CD3 (1 µg/ml) in the presence or absence of IL-2 (100 u/ml). For suppression assays, 5×10^4^ FACS-sorted CD4^+^Foxp3^−^ (GFP^−^) responder T cells were plated alone or together with T cell subsets at various ratios and activated as described above. For the last 12 hrs of culture, ^3^H-thymidine (0.5 µCi) was added and its incorporation was used to assess cell proliferation. To examine Foxp3 stability, CD4^+^ T cell subsets were stimulated with plate-bound anti-CD3 (5 µg/ml) in the presence of IL-2 (100 µ/ml).

### *Leishmania* cultures and infections

*Leishmania* promastigotes were cultured as previously shown [32]. Mice were infected with 5×10^6^ stationary phase WT or GP63^−/−^ (a gift from W.R. McMaster from UBC, Vancouver) [33] *L. major* into the right hind footpad. Disease progression was assessed and presented as a delta footpad swelling calculated by subtracting the measurement of infected footpads from non-infected footpads.

### Sodium bisulfite mapping of DNA methylation

Epitect Bisulfite Kits (Qiagen) were used for bisulfite conversion of DNA as described in the manufacturer's manual. Briefly, samples were prepared by performing nested PCR with one of the nested primers carrying a 5′ biotin modification. Primers (IDT Technologies) designed against bisulfite-converted DNA and targeting the TSDR locus were: outside – TTGAAGATTTAAGGGGGTTTTAAT (forward), ACAAATAATCTACCCCACAAATT (reverse); nested – GGTTTTTTTGGTATTTAAGAA AGA (forward), biotinylated – CAAATAATCTACCCCACAAATTTC (reverse). PCR conditions consisted of initial denaturation/enzyme activation at 95°C for 3 min, 40 cycles of 95°C for 30 sec with a respective annealing temperature of 72°C for 30 seconds, and completed with a final extension step at 72°C for 4 minutes. Pyro Sequencing was then performed using a PyroMark Q24 machine according to the manufacturer's manual. Briefly, nested PCR products were incubated with sepharose beads (GE Healthcare) and agitated for 5 minutes, then washed in 70% ethanol, denatured in 0.2 M NaOH and mixed with an annealing solution containing the relevant sequencing primers: region 1 – TTGGTATTTAAGAAAGATAG and region 2 – TAT TATTTTATTTGGGTTTA. The samples were then processed by the Pyro Sequencer, and the resulting percentage methylation at the targeted CpG sites calculated with the accompanying software (PyroMark® Q24 Software).

### Statistical analysis

Analyses were performed with a Student's *t* test. Values of *p*<0.05 were considered significant.

## Results

### Loss of Foxp3 expression in thymic or peripheral T_REG_ cells in lymphopenic hosts is modulated by the frequency of T_EFF_ but not T_REG_ cells

**Figure 1:**
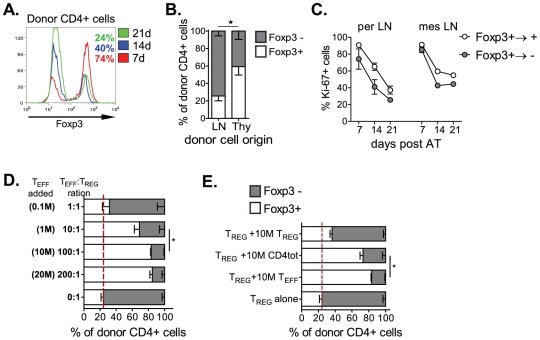
Loss of Foxp3 expression in thymic or peripheral T_REG_ cells in lymphopenic hosts is modulated by the frequency of T_EFF_ cells, not T_REG_ cells. (**A–C**) TCRβ^−/−^ mice received GFPtg CD4^+^CD25^+^ T_REG_ cells (0.3×10^6^), and 7, 14 and 21 days post transfer, donor GFP^+^ T cells from mesLN were examined for Foxp3 expression. Representative histograms of Foxp3 expression and percentage of Foxp3^+^ cells within GFP^+^CD4^+^ T_REG_ cells (**A**) and proportion of cycling cells (Ki-67 expression) within Foxp3^+^ or Foxp3^−^ donor GFP^+^CD4^+^ T cells (**C**) at various time points are shown. (**B**) TCRβ^−/−^ mice received either thymus- or LN- derived GFP^+^CD4^+^CD25^+^ T_REG_ cells. The percentage of Foxp3^+/−^ cells within GFP^+^CD4^+^ T cells is shown 14 days post adoptive transfer. (**D–E**) Recipients received GFPtg CD4^+^CD25^+^ T_REG_ cells (0.3×10^6^) either alone or in combination with indicated numbers of CD4^+^CD25^−^ T_EFF_, CD4^+^CD25^+^ T_REG_ or total CD4^+^ T cells. 14 days post T cell transfer cells, mesLN were analyzed for Foxp3 by flow cytometry. The percentage of Foxp3^+^ or Foxp3^−^ cells within donor GFP^+^CD4^+^ T cells is shown. Results are representative of 2 to 4 independent experiments (n = 3–4) are shown as mean ± SEM.

To evaluate whether Foxp3 expression was stable in T_REG_ cells *in vivo*, either FACS purified (purity >98%) CD4^+^CD25^+^ T_REG_ cells from GFP transgenic C57BL/6 (GFPtg) mice or CD4^+^GFP^+^ T_REG_ cells from Foxp3^GFPki^ reporter mice were introduced into T cell-deficient TCRβ^−/−^ recipient mice. As early as 4 days post adoptive transfer, we observed a significant loss of Foxp3 expression in donor T cells (Fig. 1A). The frequency of Foxp3-negative T_REG_ cells (Foxp3^+→−^) progressively increased from 20% on day 7 to 80% on day 21 (Fig. 1A), and reached a plateau by 4 weeks. The level of Foxp3 expression in residual Foxp3^+^ cells remained unchanged suggests that the loss of Foxp3 expression in Foxp3^+→−^ cells is not gradual (Fig. 1A).

Peripheral induced T_REG_ (iT_REG_) cells have been shown to have a less stable phenotype *in vitro* [34], [35], and may represent the major source of emerging Foxp3^+→−^ T cells in the peripheral immune system [19]. We then assessed whether Foxp3^+^ T_REG_ cells from thymus, the primary developmental site for nT_REG_ cells, possess the potential to convert into Foxp3^−^ cells. To achieve this, we reconstituted TCRβ^−/−^ recipients with FACS purified T_REG_ cells of peripheral or thymic origin and then assessed Foxp3 expression in transferred T_REG_ cells. A significant proportion of thymus-derived T_REG_ cells lose Foxp3 expression (Fig. 1B) although the frequency of Foxp3^+→−^ cells was significantly lower compared to peripheral T_REG_ cells (40% vs 70% respectively) (Fig. 1B). The rapid emergence of Foxp3^+→−^ cells from donor Foxp3^+^ T_REG_ cells was not the consequence of an outgrowth of residual Foxp3^−^ T cells present in transferred T_REG_ cells, as deliberate seeding with 1–3% of CD4^+^Foxp3^−^ T_EFF_ cells did not change the frequency of Foxp3^+→−^ T cells (data not shown). Moreover, our data indicate that the emergence of Foxp3^+→−^ T cells was not caused by a reduced proliferative capacity of Foxp3^+^ T_REG_ cells, as the Foxp3^+→+^ cells cycled similarly to Foxp3^+→−^ T cells (Fig. 1C).

We then determined whether the degree of total immune reconstitution or the T_EFF_/T_REG_ ratio in the peripheral repertoire contributes to the loss of Foxp3 expression. To this end, we introduced FACS purified T_REG_ cells in the presence of titrated numbers of congenic Ly5.1^+^ T_EFF_ cells into TCRβ^−/−^ recipients and then assessed Foxp3 expression in transferred T_REG_ cells. Co-transfer of T_EFF_ cells significantly halts the down-regulation of Foxp3 expression in T_REG_ cells in a dose dependent manner (Fig. 1D). At a physiological 10∶1 T_EFF_/T_REG_ cell ratio, the frequency of Foxp3^+→−^ cells decreased from 70% to 30%, reaching a maximal reduction at a 100∶1 T_EFF_/T_REG_ cell ratio (Fig. 1D). The frequency of Foxp3^+→−^ T cells at 100∶1 and 200∶1 T_EFF_/T_REG_ cell ratio remained unchanged. We then asked whether the nature of the T cell subset in the reconstituted host influenced the magnitude of Foxp3 downregulation in T_REG_ cells. To gain insight into this question, GFPtg T_REG_ cells (0.3×10^6^) were transferred in TCRβ^−/−^ recipients alone or in the presence of CD4^+^CD25^−^ T_EFF_, CD4^+^CD25^+^ T_REG_ or total CD4^+^ T cells (10×10^6^ each). The presence of T_REG_ cells, in contrast to T_EEF_ cells, during immune reconstitution does not stabilize Foxp3 expression in T_REG_ cells (Fig. 1E).

Collectively, our results show that highly purified T_REG_ cells from peripheral lymphoid tissues, and particularly from thymic origin, readily down-regulate Foxp3 expression in the lymphopenic environment, a process modulated by the degree of T_EFF_ cell occupancy in the periphery.

### Foxp3^+→−^ T_REG_ cell phenotype is stable and correlates with a methylated Foxp3 promoter

**Figure 2:**
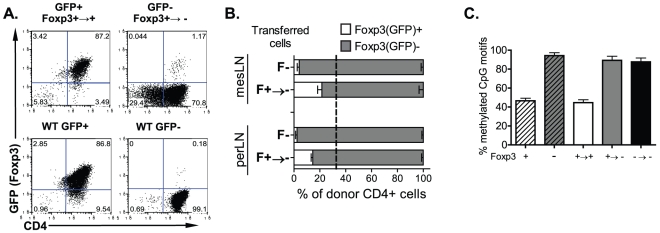
The Foxp3^+→−^ T_REG_ cell phenotype is stable and correlates with a methylated Foxp3 promoter. TCRβ^−/−^ mice received CD4^+^GFP^+^ T cells (0.5×10^6^) from Ly5.1^+^ congenic Foxp3^GFP^ reporter mice. Ly5.1^+^CD4^+^GFP^+^ (Foxp3^+^) or GFP^−^ (Foxp3^−^) cells were sorted from lymphoid tissues of recipient mice 14 days post transfer. (**A**) Sorted populations were activated *in vitro* with plate-bound anti-CD3 for 5 days or (**B–C**) re-introduced into secondary TCRβ^−/−^ recipient mice. Freshly-isolated CD4^+^GFP^+/−^ T cells from Ly5.1^+^Foxp3^GFP^ mice were used as controls. 14 days post secondary transfer, donor Ly5.1^+^CD4^+^ T cells from lymphoid tissues of secondary recipient mice were (**B**) re-analyzed for Foxp3 expression or (**C**) sorted according to GFP expression and total genomic DNA was subjected to methylation analysis of Foxp3 promoter region. The percentage of Foxp3^+^ or Foxp3^−^ cells within indicated donor T cell populations is shown in (**B**) as mean ± SEM (n = 3). (**C**) For each donor T cell population the percentage of methylated CpG motifs within Foxp3 promoter region was examined at eight different sites and averaged.

We then sought to determine whether the Foxp3^+→−^ T cell phenotype is stable *in vitro* and *in vivo*. To this end, we FACS purified Foxp3^+→−^ and Foxp3^+→+^ T cells from recipient mice, and then examined the level of Foxp3 expression after *in vitro* TCR re-stimulation under undifferentiated conditions (Fig. 2A). Neither of the fractions showed a significant change in their phenotype after 4 days of culture (Fig. 2A). Although Foxp3^+→−^ T cells re-acquired some Foxp3 (GFP) expression, this induction was nonetheless minor (1–3%) in this system.

In order to assess the stability of their phenotype *in vivo*, Foxp3^+→−^ and Foxp3^+→+^ T cells were isolated from the first recipients and re-transferred into secondary TCRβ^−/−^ recipients. Freshly-isolated Foxp3^+^ and Foxp3^−^ T cells from Foxp3^GFPki^ mice were used as control cells. Upon secondary adoptive transfer, the majority of Foxp3^+→+^ cells lose their Foxp3 expression (data not shown), similarly to freshly-isolated T_REG_ cells, suggesting that the capacity to convert into conventional T cells is a universal feature of total Foxp3^+^ T_REG_ cells rather than the unique potential of a distinct Foxp3^+^ T cell subset as was recently suggested [19]. Interestingly, 15–20% of Foxp3^+→−^ T cells re-expressed Foxp3 in different secondary lymphoid tissues examined, a percentage that is greater than the frequency of Foxp3^+^ cells (∼5%) in recipients reconstituted with freshly-isolated T_EFF_ cells (Fig. 2B). These data indicate that the Foxp3^+→−^ cell population has a stable phenotype *in vivo*, although a subset of these cells can regain Foxp3 expression.

The establishment of the stable Foxp3^+^ T_REG_ cell lineage requires selective demethylation of CpG motifs in an evolutionarily conserved element within the *foxp3* locus named TSDR (T_REG_-specific demethylated region) [15], [36], and this epigenetic imprinting in the TSDR is completely lost in Foxp3^−^ T_EFF_ cells. To establish whether Foxp3^+→−^ T cell fraction demonstrate different epigenetic modifications compared to naive Foxp3^−^ or Foxp3^+→+^ T cells, we performed bisulphite sequencing of eight evolutionarily conserved CpG motifs within the TSDR of different T cell population isolated from recipient mice following adoptive T cell transfer (Fig. 2C), as reported previously [36]. Importantly, our data shows that all eight CpG motifs of the TSDR are methylated in Foxp3^+→−^ T cells (data not shown), in contrast to Foxp3^+→+^ T cells isolated from the same recipient mice. The total TSDR methylation status (averaged from eight different sites) resembles that of freshly-isolated Foxp3^−^ T_EFF_ cells or *in vivo* activated Foxp3^−→−^ T_EFF_ cells (Fig. 2C), confirming that Foxp3 promoter methylation underlies the stable phenotype of Foxp3^+→−^ T_REG_ cells *in vivo*.

### Foxp3^+→−^ T cells lose their T_REG_ cell phenotype and reprogram into Th1 and Th17 effector cells in lymphopenic hosts

**Figure 3:**
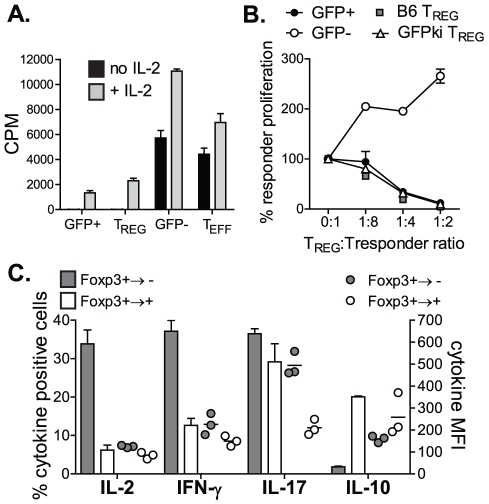
Foxp3^+→−^ T cells lose their T_REG_ cell phenotype and reprogram into Th1 and Th17 effector cells in lymphopenic hosts. (**A–B**) TCRβ^−/−^ mice received CD4^+^GFP^+^ T_REG_ cells (0.5×10^6^) isolated from Foxp3^GFP^ reporter mice, and 14 days later, donor CD4^+^GFP^+^(Foxp3^+^)/GFP^−^(Foxp3^−^) cells were sorted from recipient mice and their proliferation (**A**) and suppressive activity (**B**) were assessed following *in vitro* activation. Freshly isolated T_REG_ and T_EFF_ cells from Foxp3^GFP^ mice were used as controls. Data from one of three independent experiments is presented as mean ± s.d. of triplicate wells. (**C**) TCRβ^−/−^ mice received GFPtgCD4^+^CD25^+^ T_REG_ cells (0.3×10^6^), and 14 days post transfer, GFP^+^ donor T cells were examined for the production of various cytokines relative to Foxp3 expression. Frequencies and mean fluorescent intensity (MFI) (**C**) of cytokines produced by Foxp3^+/−^ cells are shown as mean ± SEM from one out of 4 independent experiments (n = 4).

We then investigated whether the loss of Foxp3 led to a deficiency in T_REG_ cell function. To this end, we tested their capacity to respond to TCR-induced proliferation and suppressive function *in vitro*. Foxp3^+→−^ T cells proliferated *in vitro* with a slightly greater rate than freshly-isolated T_EFF_ cells even in the absence of exogenous IL-2 (Fig. 3A). In contrast, Foxp3^+→+^ cells retained their unresponsiveness to TCR-induced proliferation (anergy), and proliferated only in the presence of exogenous IL-2 (Fig. 3A). More importantly, Foxp3^+→−^ cells completely lost their suppressive function *in vitro* in contrast to Foxp3^+→+^ cells, which efficiently suppressed proliferation of responding T_EFF_ cells (Fig. 3B). Furthermore, adoptive transfer of Foxp3^+→−^ cells into lymphopenic hosts led to a significant lymphocytic infiltration in the colon in contrast to the Foxp3^+→+^ cell transfer (data not shown). These results indicate that Foxp3^+→−^ T cells lose the *bona fide* T_REG_ phenotype, and gain the behaviour of conventional T_EFF_ cells *in vitro* and *in vivo*.

To further examine whether Foxp3^+→−^ cells can acquire functional properties of T_EFF_ cells, LN cells from TCRβ^−/−^ recipient mice reconstituted with FACS-purified Foxp3^+^ T_REG_ cells were re-stimulated *ex vivo* with PMA and ionomycin and analyzed for the production of pro-inflammatory cytokines relative to Foxp3 expression (Fig. 3C). We show that over 30% of Foxp3^+→−^ T cells produced high levels of IL-2, IFN-γ and IL-17 and show a decreased production of IL-10 in contrast to Foxp3^+→+^ cells which show low frequencies of IL-2 and IFN-γ secreting T cells (<5% and 10% respectively). Although the frequency of IL-17-producing cells within stable Foxp3^+→+^ T_REG_ cell fraction was comparable to Foxp3^+→−^ T cells (up to 30%), the magnitude of cytokine expression (i.e. MFI) was significantly lower in Foxp3^+→+^ cells (Fig. 3C). Thus, Foxp3 down-regulation in committed T_REG_ cells impacts their cell fate and differentiation, and forces their reprogramming into Th1 and Th17 effector cells in lymphopenic hosts.

### Intestinal inflammation or parasitic infection favours the reprogramming of Foxp3^+^ T_REG_ cells into effector T cells and promotes host immunity

**Figure 4:**
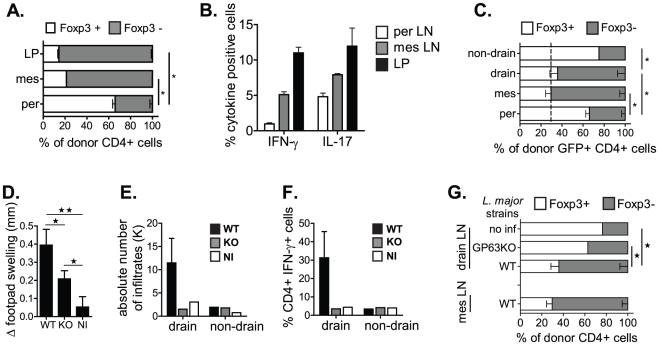
Intestinal inflammation or parasitic infection favours the reprogramming of Foxp3^+^ T_REG_ cells into effector T cells and promotes host immunity. (**A–B**) TCRβ^−/−^ mice received GFPtg CD4^+^CD25^+^ T cells (0.3×10^6^). 14 days later Foxp3 expression within donor GFP^+^CD4^+^ T cells (**A**) and the frequency of total IFN-γ or IL-17 producing cells (**B**) in indicated tissues of recipient mice from one of 3 representative experiments (n>3) is shown. (**C–G**) TCRβ^−/−^ mice were infected or not (NI) with 5×10^6^ promastigotes of WT or GP63^−/−^ (KO) *L. major* into the right footpad 2 weeks prior reconstitution with GFPtgCD4^+^CD25^+^ T_REG_ cells (0.3×10^6^). (**C**) 4 weeks later, GFP^+^CD4^+^ T cells from draining (infected) and non-draining popliteal LN, perLN and mesLN were analyzed for Foxp3 expression. Footpad swelling (**D**), absolute number of infiltrated lymphocytes (**E**), and frequencies of IFN-γ producing CD4^+^ T cells (**F**) are shown in infected and non-infected sites. (**G**) The loss of Foxp3 expression by T_REG_ cells was compared between mice infected with WT or GP63^−/−^ *L. major* strains. Results are representative of 2 independent experiments with n = 4–5.

We then compared Foxp3 expression in donor T_REG_ cells from different lymphoid tissues. We observed a 2–3 fold reduction in the conversion of Foxp3^+^ T_REG_ cells into Foxp3^−^ T cells in perLN compared to mesLN of recipient mice (20–30% vs 80%) (Fig. 4A). Furthermore, the greatest Foxp3 down-regulation in donor T_REG_ cells was observed in LP as the frequency of Foxp3^+→−^ T cells in this site reached 90% (Fig. 4A). These data suggest that the intestinal microenvironment is conducive for the conversion of Foxp3^+^ T_REG_ cells into conventional Foxp3^−^ T cells. A distinct characteristic of mucosal tissues is the constant stimulation of the local immune system with bacterial antigens. Notably, TCRβ^−/−^ mice are known to manifest spontaneous inflammation specifically in the intestine in the absence of any immune reconstitution. Moreover, this inflammatory response is known to be driven by the intestinal flora [37]. The analysis of inflammatory cytokines at the different sites upon T_REG_ cell transfer shows a 2- and 8-fold increase of total IL-17 and IFN-γ secretion, respectively, in mucosa associated tissues compared to perLN (Fig. 4B). This suggests that the increased production of inflammatory cytokines in the gut correlates directly with an increased down-regulation of Foxp3 in donor T_REG_ cell population in mesenteric sites.

To directly assess the role of local, microbial-induced inflammation in T_REG_ cell reprogramming, we used a model of cutaneous *L. major* infection. To this end, we inoculated intradermally *L. major* promastigotes into the right hind footpad of TCRβ^−/−^ mice, and adoptively transferred GFPtg T_REG_ cells 2 weeks post-infection. While control non-infected popliteal LN (popLN) show a similar frequency of Foxp3^+→−^ T cells compared to perLN in infected mice (∼20%), we observe a dramatic increase in Foxp3^−^ T cells in infected draining LN as 65% of donor T_REG_ cells down-regulated Foxp3 expression (Fig. 4C). Interestingly, *L. major* infected sites show a comparable frequency of Foxp3^+→−^ cells compared to mesLN (Fig. 4C) suggesting that microbial-induced inflammation drives T_REG_ cell reprogramming.

We then determined whether the level of microbial-induced inflammation in the local T_REG_ environment influences the extent of T_REG_ cell reprogramming. To achieve this, we compared Foxp3 expression in donor T_REG_ cells in recipient mice infected either with a WT strain of *L. major* or a mutant strain lacking GP63 (GP63^−/−^), a key virulence factor directly involved in parasite-host interactions, and promoting Th1 immune responses in the infected host. Thus, GP63^−/−^ *L. major* are less infectious and trigger attenuated inflammatory responses *in vivo*.

Infection with GP63^−/−^ *L. major* resulted in reduced footpad swelling compared to WT strain (Fig. 4D) and resulted in minimal leukocyte infiltration and IFN-γ production, which was comparable to non-infected recipients (Fig. 4E, F). We observed that the frequency of Foxp3^+→−^ GFP^+^ T cells in draining popLN of GP63^−/−^ *L. major* infected mice was 2-fold lower compared to WT *L. major* infected recipients (35% vs 70% respectively) and slightly higher compared to non-infected recipients (35% vs 20% respectively) (Fig. 4G). In addition, we show that the increased magnitude of Foxp3 down-regulation correlates with a significantly higher proportion of IFN-γ secreting T cells in *L. major* infected sites, in stark contrast to non-infected LN (Fig. 4F) demonstrating that attenuated inflammatory conditions result in significantly reduced Foxp3 loss in donor T_REG_ cells. Overall, our results show a direct, magnitude-dependent role of microbial-induced inflammatory signals in the down-regulation of Foxp3 expression and reprogramming in donor T_REG_ cells.

### T_REG_ cell reprogramming occurs independently of variations in IL-2 production in vivo

**Figure 5:**
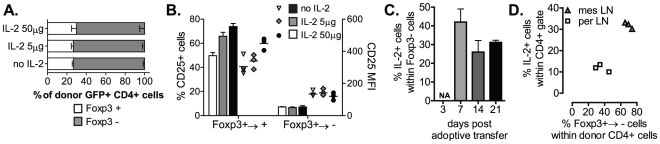
T_REG_ cell reprogramming occurs independently of variations in IL-2 production *in vivo*. (**A–B**) TCRβ^−/−^ mice were treated every other day with 5 or 50 ng of rhIL-2, starting 2 days prior transfer of GFPtg CD4^+^CD25^+^ T cells (0.3×10^6^). MesLN were analyzed for Foxp3 expression 14 days later. The percentage of Foxp3^+/−^ cells (**A**) and the frequency of CD25^+^ cells and MFI of CD25 expression (**B**) within donor GFP^+^CD4^+^ T cells are shown. (**C–D**) TCRβ^−/−^ mice received GFPtg CD4^+^CD25^+^ T cells (0.3×10^6^). Donor T cells from perLN and mesLN were analyzed for Foxp3 expression and IL-2 production. (**C**) The frequency of IL-2 secreting GFP^+^CD4^+^Foxp3^−^ T cells and (**D**) correlation between frequency of IL-2^+^ and Foxp3^−^ cells within donor GFP^+^CD4^+^ T cells is shown. Results are representative of 2 independent experiments (n = 3–4).

One critical factor responsible for the development, function and homeostasis of Foxp3^+^ T_REG_ cells is IL-2, a cytokine primarily produced by activated T_EFF_ cells. Fluctuations in the bio-availability of IL-2 in inflammatory sites may perturb the T_REG_/T_EFF_ balance in these sites and trigger autoimmunity [38]–[41]. Duarte *et al.* [20] recently showed that prophylactic IL-2 infusion *in vivo* can prevent Foxp3 down-regulation in T_REG_ cells suggesting that a temporal deficiency in IL-2, possibly as a consequence of the absence of T_EFF_ cells, was the initial trigger for Foxp3 loss in T_REG_ cells. However, in our system, the delivery of high (50 ng) or low (5 ng) doses of rhIL-2, previously shown to be favourable for the homeostasis of Foxp3^+^ T_REG_ cells and protection from autoimmune diabetes [40], failed to prevent the loss of Foxp3 expression in donor T_REG_ cells in all tissues examined (Fig. 5A). Moreover, the time or frequency of treatment initiation did not change the outcome (data not shown). Interestingly, both low and high IL-2 dose treatments resulted in a significant increase in CD25 expression (MFI) and frequency of CD25^+^ cells solely within the stable Foxp3^+→+^ T cell fraction but not in Foxp3^+→−^ cells (Fig. 5B). This suggests that Foxp3^+→+^ T cells may be particularly sensitive to IL-2 signals *in vivo*.

We then assessed how the production of IL-2 *in vivo* related with the onset and magnitude of Foxp3 loss in donor T_REG_ cells. Our earlier results show that unlike stable Foxp3^+→+^ T_REG_ cells, around 30% of Foxp3^+→−^ T cells in mesenteric LN (mesLN) secrete IL-2 (Fig. 3C) as soon as they are detected in the system (Fig. 5C). Interestingly, we show a 2-fold increase in the frequency of IL-2^+^ Foxp3^+→−^ T cells in mesLN compared to perLN (Fig. 5D) despite the more significant loss of Foxp3 in donor T_REG_ cells in mucosa-draining LN. Thus, the magnitude of Foxp3 loss in donor T_REG_ cells inversely correlated with the frequency of IL-2 secreting Foxp3^+→−^ CD4^+^ T cells. Overall, these results indicate that Foxp3 down-regulation in donor T_REG_ cells is not triggered solely by a deficiency in T cell-derived IL-2 in secondary lymphoid tissues.

### Predominant IL-17 secretion precedes Foxp3 down-regulation in reprogramming Foxp3^+^ T_REG_ cells, a process accentuated in mesenteric sites

**Figure 6:**
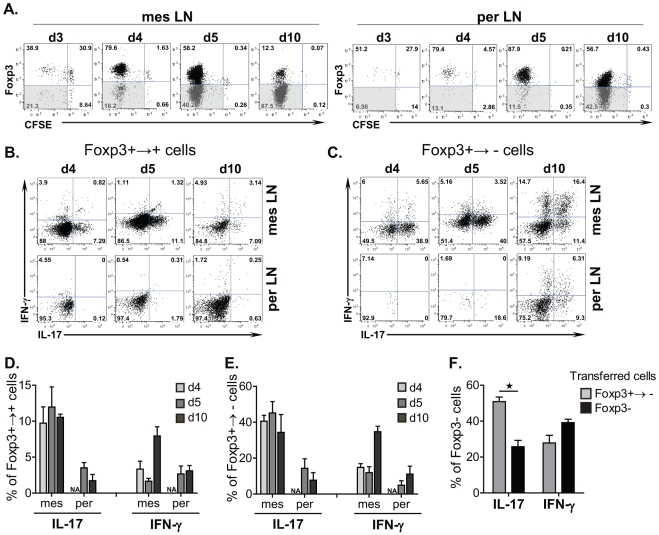
Predominant IL-17 secretion precedes Foxp3 down-regulation in reprogramming Foxp3^+^ T_REG_ cells, a process accentuated in mesenteric sites. (**A–E**) TCRβ^−/−^ mice received CFSE-labelled CD4^+^CD25^+^ T cells (0.3×10^6^) isolated from congenic Ly5.1^+^ mice, and, donor T cells were examined for Foxp3 expression at the indicated timepoints post transfer. (**A**) Representative FACS plots of Foxp3 expression relative to CFSE dilution in donor Ly5.1^+^CD4^+^ T cells are shown at various time points. (**B–E**) Representative FACS profiles (**B,C**) and proportion (**D,E**) of IL-17/IFN-γ-secreting donor Ly5.1^+^CD4^+^Foxp3^+^ (**B,D**) and Foxp3^−^ (**C,E**) T cells undergoing expansion at various time points are shown. Results are representative of 2 independent experiments (n = 3). (**F**) TCRβ^−/−^ mice received CD4^+^GFP^+^ T cells (0.5×10^6^) isolated from Ly5.1^+^ congenic Foxp3^GFP^ reporter mice, 14 days post transfer Ly5.1^+^CD4^+^GFP^−^ cells (Foxp3+→−) were sorted from lymphoid tissues of recipient mice, and reintroduced into secondary TCRβ^−/−^ recipient mice. Freshly-isolated CD4^+^GFP^−^ T cells (Foxp3-) from Ly5.1^+^Foxp3^GFP^ mice were used as a control. 14 days post secondary transfer, donor Ly5.1^+^CD4^+^ T cells from mesLN of secondary recipients were analyzed for IL-17/IFN-γ secretion relative to Foxp3 expression. Proportion of cytokine producing Foxp3^−^Ly5.1^+^CD4^+^ T cells is shown as mean ± SEM from one out of 2 independent experiments (n = 4).

To assess whether cellular expansion contributed to Foxp3 down-regulation in T_REG_ cells, the level of Foxp3 expression in donor CFSE-labelled CD4^+^CD25^+^ T_REG_ cells was determined 3, 4, 5 and 10 days post adoptive cell transfer (Fig. 6A). Our analysis shows that Foxp3^+→−^ T cells emerge already by 3 days post T cell transfer in the mesLN, and Foxp3 down-regulation occurs only in actively proliferating T cells (Fig. 6A). Interestingly, we observed a delay in the emergence of Foxp3^+→−^ T cells in perLN compared to mesLN, although the proportion of divided donor T cells in both lymphoid tissues was similar (51% vs 39% at day 3 and 80% vs 80% at day 4) (Fig. 6A). This finding strongly suggests that Foxp3 down-regulation in donor T_REG_ cells is only partly attributed to the homeostatic proliferation during lymphopenia.

A kinetic analysis of cytokine production by Foxp3^+→−^ and Foxp3^+→+^ T cells revealed prominent IL-17 secretion, which temporally preceded IFN-γ production in both fractions of donor T_REG_ cells (Fig. 6B–E). The predominant IL-17 secretion was particularly marked in Foxp3^+→−^ T cells in mesLN (Fig. 6C, E). By 4 days post adoptive transfer, 40% of Foxp3^+→−^ T cells secreted IL-17 and only 10% secreted IFN-γ. Interestingly, while the proportion of IFN-γ producing Foxp3^+→−^ and Foxp3^+→+^ T cells increased with time, reaching respectively 30% and 10% at day 10, the proportion of IL-17^+^ cells did not vary (Fig. 6C, E), suggesting a bias for IL-17-polarization in reprogrammed Foxp3^+→−^ T cells.

To establish whether Th17-like Foxp3^+→−^ T cells display a phenotype reminiscent of conventional Foxp3^−^ T_EFF_ cells, the cytokine profile of Foxp3^+→−^ and Foxp3^−→−^ T cell populations was determined upon secondary adoptive T cell transfer into new TCRβ^−/−^ recipients. Our data show that 14 days post secondary T cell transfer, re-programmed Foxp3^+→−^ T cells maintained their phenotype and contained 2-fold higher proportion of IL-17 producing cells compared to Foxp3^−→−^ T cells (Fig. 6F). Interestingly, Foxp3^+→−^ T cells still produced a comparable amount of IFN-γ compared to conventional Foxp3^−^ T_EFF_ cells (Fig. 6F), suggesting that the re-programmed Foxp3^+→−^ T cells display a more pro-inflammatory IL-17-dominant phenotype.

### mTOR inhibition stabilizes Foxp3 expression in T_REG_ cells and strongly inhibits IL-17 but not ROR-γt expression in vivo

**Figure 7:**
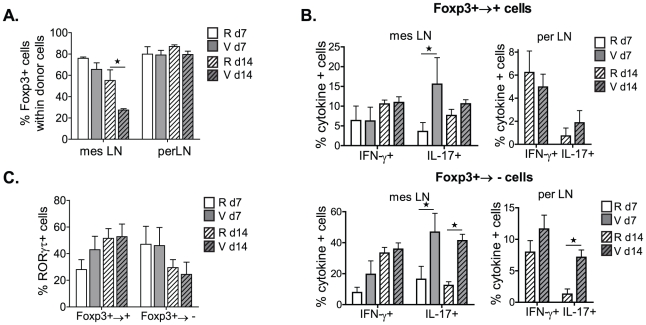
mTOR inhibition stabilizes Foxp3 expression in T_REG_ cells and strongly inhibits IL-17 but not RORγt expression. TCRβ^−/−^ mice were transferred with GFPtg CD4^+^CD25^+^ T_REG_ cells (0.3×10^6^), and then treated every second day with rapamycin (R) (18 mg/kg) or control vehicle (formulation without rapamycin) (V) as of day 0. 7 and 14 days later, perLN and mesLN of recipient mice were analyzed by FACS. (**A**) Foxp3 expression within donor GFP^+^CD4^+^ T cells, (**B**) proportion of IL-17/IFN-γ producing Foxp3^+/−^ donor T cells and (**C**) RORγt expression within Foxp3^+/−^ donor T cells are shown as mean ± SD from one out of 2 independent experiments (n = 4).

Rapamycin is an immunosuppressive drug which inhibits the mTOR signalling pathway and selectively promotes Foxp3^+^ T_REG_ lineage differentiation *in vitro* and *in vivo*, while inhibiting the differentiation of Foxp3^−^ T_EFF_ cells [25], [42]. In order to examine whether Foxp3 down-regulation in donor T_REG_ cells is mTOR pathway dependant, TCRβ^−/−^ recipient mice were treated with rapamycin every other day after Foxp3^+^ T_REG_ cell transfer, and donor T cells from mesLN and perLN were analyzed for Foxp3 expression 7 and 14 days later. Our data show that while rapamycin did not have any effect on the level of Foxp3 expression in donor T_REG_ cells in perLN, it potently inhibited Foxp3 down-regulation in mesLN 14 days post adoptive transfer, consequently rescuing the proportion of Foxp3^+^ donor T_REG_ cells from 27% up to 55% (Fig. 7A). We also observed a significant decrease in IL-17-producing donor T cells following *in vivo* rapamycin treatment, whereas the frequency of IFN-γ secreting T cells remained unchanged (Fig. 7B). Reprogrammed Foxp3^+→−^ T cells in both perLN and mesLN showed the most marked decrease (4-fold) in the proportion of IL-17-secreting T cells (Fig. 7B). These results are consistent with previous studies illustrating rapamycin-mediated inhibition of Th17 cells [28].

As RORγt is a critical factor for Th17 cell development, we hypothesized that inhibition of the mTOR pathway would prevent IL-17 secretion by abrogating RORγτ expression. Surprisingly, rapamycin treatment did not alter RORγt expression either in re-programmed Foxp3^+→−^ or in stable Foxp3^+→+^ T cells (Fig. 7C), suggesting that inhibition of the mTOR pathway selectively influences other factors involved Th17 cell differentiation. Thus, rapamycin-mediated inhibition of mTOR activity stabilizes the Foxp3^+^ T_REG_ cell pool, particularly in inflammatory mesenteric sites, and strongly inhibits Th17 cell development *in vivo*.

## Discussion

It is commonly viewed that Foxp3-expressing T cells represent a stable, terminally-differentiated lineage responsible for suppression of a wide variety of immune responses and maintenance of peripheral self-tolerance. Recently, studies proposed that Foxp3^+^ T_REG_ cells, or an unstable subpopulation found within, retains developmental plasticity, whose modulating factors have yet to be clearly elucidated [19]–[21].

Here, we show that thymic and peripheral Foxp3^+^ T_REG_ cells of normal, unmanipulated mice possess the potential to lose Foxp3 expression *in vivo*. This reprogramming of T_REG_ cells provokes the loss of the *bona fide* Foxp3^+^ T_REG_ cell phenotype and re-directs their effector differentiation primarily to Th1 and Th17 cell lineages. Further *in vitro* and *in vivo* comparison of Foxp3^+→−^ and naïve Foxp3^−^ T_EFF_ cells revealed the strong similarities in their phonotypical and functional characteristics as well as epigenetic modifications of Foxp3 promoter region. The fact that Foxp3^+→−^ T cells produce more IL-17 upon secondary adoptive transfer into lymphopenic host compared to naïve Foxp3^−^ T cells, suggests a possible bias for Th17 polarization in unstable Foxp3^+→−^ T_REG_ cells. Moreover, our data propose that IL-17^+^Foxp3^+^ T cells, which are found in secondary lymphoid organs of recipient mice, may possibly represent a transitional stage of Foxp3^+^ T_REG_ cells converting to Foxp3^+→−^ T cells.

We show that reprogrammed Foxp3^+→−^ T_REG_ cells manifest a stable phenotype following TCR re-activation in un-polarized conditions *in vitro* and after extensive cell division in lymphopenic hosts. This is in contrast to a recent study showing that reprogrammed Foxp3^+→−^ T_REG_ cells have the potential to reacquire Foxp3 expression under TGF-β1 inducing conditions [19]. Only around 20% of Foxp3^+→−^ T cells regain Foxp3 expression suggesting that TCR re-activation in Foxp3^+→−^ T cells does not re-establish the “memory” of original Foxp3 expression in parent cells by enabling *de novo* active transcription at the remodelled *foxp3* locus [15].

We show that while Foxp3 loss is primarily seen in actively dividing T_REG_ cells in lymphopenic mice, the pre-existing T_EFF_/T_REG_ ratio is an important variable in Foxp3^+→−^ T cell generation. We observe that the increased frequency of total or effector CD4^+^ T cells co-transferred with T_REG_ cells reduces the magnitude of the loss of Foxp3 expression in T_REG_ cells. The minimal loss of Foxp3 expression (15%), achieved at high 100∶1 T_EFF_/T_REG_ ratio, correlates with the basal level of the Foxp3 down-regulation detected in normal lymphoreplete mice [22], suggesting that the degree of total reconstitution in lymphopenic hosts prevents Foxp3 loss. Moreover, co-transfer of similar numbers of T_REG_ cells does not modulate the level of Foxp3 down-regulation, suggesting that the total degree of Foxp3 loss may correlate with a lack of occupancy of T_EFF_ cell niches or T_EFF_ derived signals.

The particular T_EFF_ cell-mediated signals that stabilize Foxp3 expression are still unknown. IL-2, a likely candidate, was recently shown to prevent Foxp3 down-regulation in T_REG_ cells suggesting that a temporal deficiency in IL-2, possibly as a consequence of the absence of T_EFF_ cells in the periphery, was the initial trigger for Foxp3 loss in T_REG_ cells [20]. Several lines of evidence from our study do not support a causative role for an IL-2 deficiency in the generation of Foxp3^+→−^ T cells. We show that the magnitude of Foxp3 loss in donor T_REG_ cells inversely correlated with the frequency of IL-2 secreting Foxp3^+→−^ CD4^+^ T cells suggesting that Foxp3 down-regulation is not triggered solely by a deficiency in T cell-derived IL-2 in secondary lymphoid tissues. Moreover, both low and high dose prophylactic IL-2 treatments resulted in a significant increase in CD25 expression and frequency of CD25^+^ cells only within the stable Foxp3^+^ T cell fraction suggesting that IL-2 preferentially favours the fitness of stable Foxp3^+→+^ T_REG_ cells without preventing T_REG_ cell reprogramming.

The greatest degree of T_REG_ cell plasticity was observed in the gut microenvironment, and reprogramming of T_REG_ cells associated with significantly higher production of pro-inflammatory cytokines, particularly IL-17, in mesenteric sites. The more elevated basal inflammation induced by the constant exposure to commensal microbes coincides with the magnitude of Foxp3^+→−^ cell reprogramming. This correlates with the recently published study indicating the gastrointestinal tract is a specific site for generation and control of Th17 cells [43]. Moreover, the locally induced infection with WT or attenuated form of *L. major* directly confirms that the magnitude of pathogen-induced inflammation is a critical factor in the emergence of T_EFF_ cells from Foxp3^+^ T_REG_ cell population. The possibility of concomitant recruitment of reprogrammed Foxp3^+→−^ cells to inflammatory sites from other lymphoid sites cannot be excluded. While the nature of the innate signals remain unidentified, the substantial emergence of IL-17 producing cells in both Foxp3^−^ and Foxp3^+^ fractions of donor T_REG_ cells indicates the involvement of Th17 promoting factors in generation of Foxp3^+→−^ T cells. *In vitro*, IL-6 inhibits Foxp3 expression *in vitro* [18] and, in combination with TGF-β, promotes the generation of IL-17 producing cells from conventional [44], [45] or regulatory T cells [16], [17]. Recently, it was shown that *L. major* infection triggers IL-6 secretion in DC [46] and keratinocytes [47], and that IL-6 neutralization, together with an exacerbated *L. major*-induced pathology, increased local T_REG_ cell numbers in the site of infection [48]. Thus, these data suggest a role for Th17 polarizing factors not only in anti-parasitic immunity but also in the reprogramming of T_REG_ cells into Foxp3^−^ T_EFF_ cells.

The PI3K/Akt pathway primarily signals through the mTOR, a master regulator that integrates metabolic, environmental and inflammatory cues, which ultimately promotes cell growth, proliferation, and T cell differentiation. More specifically, PI3K/Akt/mTOR signalling abrogates T_REG_ cell differentiation, and over-expression of an active form of Akt abrogates TGF-β-induced Foxp3 expression in CD4^+^ T cells [26], [49]. Our results demonstrate that mTOR inhibition by rapamycin, prevents Foxp3 down-regulation, stabilizes the Foxp3^+^ T_REG_ cell pool, particularly in the inflammatory gut microenvironment, and strongly inhibits T_REG_ cell reprogramming into Th1 and Th17 effectors *in vivo*. These results are consistent with previous studies showing that similar inhibition of mTOR signalling, either through genetic deletion or rapamycin treatment, promotes T_REG_ differentiation while blunting T_EFF_ cell differentiation and function *in vitro* and *in vivo* [25], [27]. Furthermore, we show that Foxp3^+^ T_REG_ cells readily express significant levels of RORγt, as confirmed by previous studies [50], [51], and indicates that rapamycin-mediated inhibition of IL-17 production by re-programmed Foxp3^+→−^ T cells does not correlate with a reduction in RORγt expression and may selectively influence other factors involved Th17 cell differentiation. Overall, our results suggest that changes in metabolic, inflammatory or environmental signals within the T_REG_ cell microenvironment modulate mTOR function, and may impact the stability of the Foxp3^+^ expression and T_REG_ cell function in settings of tolerance or immunity.

A lingering, controversial question concerns the cellular origin of newly-generated Foxp3^+→−^ T cells. Recently, a study by Komatsu and colleagues [19] suggests that only a small fraction of Foxp3^+^ nT_REG_ cells, negative for CD25 expression, possesses the ability to convert into Foxp3^−^ T cells in contrast to the Foxp3^+^CD25^+^ cell subset representing a stable T_REG_ population. In our study we cannot attribute the down-regulation of Foxp3 to any particular subpopulation of nT_REG_ cells since both CD4^+^CD25^high^ (excludes CD25^Neg^Foxp3^+^) and CD4^+^Foxp3^GFP+^ (includes CD25^Neg^Foxp3^+^ and CD25^+^Foxp3^+^ subsets) subpopulations demonstrate similar ability to lose Foxp3 expression *in vivo*. Moreover, the observation that a significant proportion of the stable Foxp3^+^ donor T cells continued to down-regulate Foxp3 expression upon secondary adoptive transfer strongly suggests that the total Foxp3^+^ T_REG_ population rather than a specific fraction exhibits this functional plasticity.

Overall, the findings of this study highlight the dynamics of Foxp3 expression in committed Foxp3^+^ T_REG_ cells, and point to the nature and magnitude of inflammation as critical factors modulating the plasticity of Foxp3^+^ T_REG_ cells. As peripheral Foxp3^+^ T_REG_ cells display an augmented potential for Foxp3 down-regulation compared to thymus-derived Foxp3^+^ T cells, caution should be taken in designing future therapeutic studies involving T_REG_ cell infusions. However, as no inflammation or disease induction was observed in T_REG_ cell-reconstituted recipient mice even with a maximum degree of Foxp3 down-regulation, this may suggest that the consequence of this T_REG_ cell plasticity is to ensure the occupancy of the conventional T cell niche by Foxp3^+→−^ T cells in the lymphopenic environment and maintain a T_EFF_/T_REG_ equilibrium in the peripheral immune system.
